# Mapping the ecological dimensions and potential distributions of endangered relic shrubs in western Ordos biodiversity center

**DOI:** 10.1038/srep26268

**Published:** 2016-05-20

**Authors:** Geng-Ping Zhu, Hui-Qi Li, Li Zhao, Liang Man, Qiang Liu

**Affiliations:** 1 Tianjin Key Laboratory of Animal and Plant Resistance, College of Life Sciences, Tianjin Normal University, 393 Binshui Road, Tianjin 300387, China

## Abstract

Potential distributions of endemic relic shrubs in western Ordos were poorly mapped, which hindered our implementation of proper conservation. Here we investigated the applicability of ecological niche modeling for endangered relic shrubs to detect areas of priority for biodiversity conservation and analyze differences in ecological niche spaces used by relic shrubs. We applied ordination and niche modeling techniques to assess main environmental drivers of five endemic relic shrubs in western Ordos, namely, *Ammopiptanthus mongolicus, Amygdalus mongolica, Helianthemum songaricum, Potaninia mongolica*, and *Tetraena mongolica*. We calculated niche overlap metrics in gridded environmental spaces and compared geographical projections of ecological niches to determine similarities and differences of niches occupied by relic shrubs. All studied taxa presented different responses to environmental factors, which resulted in a unique combination of niche conditions. Precipitation availability and soil quality characteristics play important roles in the distributions of most shrubs. Each relic shrub is constrained by a unique set of environmental conditions, the distribution of one species cannot be implied by the distribution of another, highlighting the inadequacy of one-fits-all type of conservation measure. Our stacked habitat suitability maps revealed regions around Yellow River, which are highly suitable for most species, thereby providing high conservation value.

The investigation of ecological niches conservatism has become a significant research topic, together with the identification of main environmental constraints on species’ distribution, given the expected impacts of climate change on biodiversity^1^,^2^,^3^,^4^. This outcome has prompted new tools development to assess how ecological niche of a species can shrink, expand or persist, in environmental and geographic spaces while anticipating climate change effects^5^,^6^. Currently, ecological niche differences among species can be visualized and analyzed to evaluate the possible ecological and evolutionary forces that shape geographical distributions and habitat preferences^7^,^8^. Species groups that are highly diverse may present a varied set of ecological adaptations along an environmental gradient which are of importance for understanding ecological niche differences and to prepare mitigation actions against climate change impacts. Information about the potential changes in ecological niches can be used to implement or guide conservation actions, especially in biodiversity-rich areas^9^.

Various methods have been proposed to study niche conservatism, the study of how species vary in their requirements for or tolerance of these factors has advanced, in part due to the conceptual development of ecological niche^10^. Methods for quantification and estimation of niche differences typically rely on either ordination techniques^11^ or ecological niche modeling (ENM)^12^. The former approach allows direct comparisons of species–environment relationships in environmental spaces^13^, whereas the latter is widely adopted in conservation biogeography. ENM assumes that each species occupies its own particular Grinnellian niche on a macroscale^7^. This modeling technique uses occurrence-associated environmental variables to assess potential distribution^12^. Niche overlap is then estimated through model predictions across a landscape. The development of ENM has facilitated the extraction of ecological niche characteristics, which can assist in biodiversity conservation^14^.

The western Ordos plateau is a relatively independent physiographic unit in the semi-arid zone in central-northern China (Fig. 1). Climate in this area is extremely cold during winter and spring and very dry during summer and fall. Shrubs are the dominant plant life forms in this region^15^,^16^. This geographically restricted area has developed not only rich shrub diversity but also high percentage of relic species and endemic taxa^17^, being as one of the eight floristic endemic centers in China^18^. These relic shrubs belong to the Mediterranean flora, they are playing an important role in prevention of soil erosion and desertification in arid areas (Fig. 1). However, the ecological requirements and geographical distributions of these relic shrubs were poorly mapped, many scattered populations were discovered beyond historical ranges. Additionally, the western Ordos plateau is rich in mineral resources (e.g. coal, kaolin, quartzite, and ironstone). Biodiversity in this area is suffering from heavy human disturbances^17^, which predominantly include mining activities and associated refining processes, infrastructure construction (e.g. highway) associated with urbanization. Grazing has also posed a serious threat to endangered relic shrubs in this area.

In present study, we selected five endangered endemic relic shrub taxa, namely, *Ammopiptanthus mongolicus, Amygdalus mongolica, Helianthemum songaricum, Potaninia mongolica*, and *Tetraena mongolica* based on their importance in western Ordos. For example, Tetraenoideae is a monotypic subfamily from Zygophyllaceae with only one genus, which contains only one species (i.e. *Tetraena mongolica*). This single species is endemic to western Ordos plateau (Fig. 1) and defined as a rare and endangered species because of its extremely restricted distribution and very scarce population^19^. Here we analyzed how species-specific responses to environmental factors and differences in ecological niche space can aid future conservation. To this end, we used ENM and ordination techniques to characterize ecological niches of five relic shrubs and quantify the similarities between them. First, we identified main environmental variables that constrain their distributions. Second, we used information on their environmental constraints to generate consensus habitat suitability map to highlight hotspots of habitat suitability to inform conservation planning. Third, we assessed the similarities and differences of ecological spaces occupied by relic shrubs to investigate how differences in the distributions of ecological niche spaces and species-specific responses to environmental factors may inform conservation plans. Finally, we mapped the suitability of each shrub in dimensions of human disturbance to assess human threats. Similarities existed in the morphological and physiological characteristics of these shrubs. Thus, we assumed their ecological niches to be similar. However, due to the different adaptations to diverse environments, we expected ecological niches to be non-equivalent.

## Results

### Importance of environmental variables

Ten environmental variables (i.e. temperature mean diurnal range (BIO2), maximum temperature of warmest month (BIO6), aridity index (AI), aspect, compound topographic index (CTI), growing degree days (GDD), normalized difference vegetation index (NDVI), slope, soil nutrient availability (SQ1) and soil rooting conditions (SQ3) were finally used by considering biological relevance and spatial correlation (see below method for detail). Distributions of relic shrubs were underpinned by their different responses to the environment (Fig. 2). In western Ordos, shrub distributions were mainly constrained by a combination of AI, slope, and rooting conditions (Table 1). For *A. mongolicus* and *A. mongolica*, the AI and slope degree were important variables. In *A. mongolicus*, the suitability increased along BIO2, GDD, slope degree, and SQ1; however, such suitability decreased along AI and NDVI. The suitability of *A. mongolica* exhibited a unimodal response to AI, aspect, GDD, and slope degree; nonetheless, a linear response to BIO2, CTI, NDVI, SQ1, and SQ3 was observed (Fig. 2). For *H. songaricum*, soil qualities were important variables, including both SQ1 and SQ3, in which a remarkably negative response to SQ1 was observed (Fig. 2). NDVI and slope degree showed significant contribution to *P. mongolica*’s distribution, whereas NDVI and AI contributed significantly to *T. mongolica*’s distribution (Table 1). A unimodal response to AI, BIO2, and NDVI was observed in *T. mongolica* (Fig. 2). Different from expected, none of the shrubs were strongly constrained by BIO6, aspect, and CTI. Most shrubs demonstrated responses to variables that usually overpassed logistic suitability estimates of 0.5.

### Biodiversity hotspots

Models of five shrubs predicted by Maxent closely matched their known distributions (Fig. 3). These predictions obtained training AUC values of 0.90, 0.79, 0.99, 0.72, and 0.98 for *A. mongolicus, A. mongolica, H. songaricum, P. mongolica*, and *T. mongolica*, respectively (Supplementary Table 1), which strongly supported their predictive power (except for *P. mongolica*). The null model protocol suggests that our results are significantly better than those expected by a random model. In fact, all ENMs performed significantly better than expected by chance alone (*P* < 0.01; Supplementary Table 1), except *P. mongolica*. These models successfully identified three wide-range species (*A. mongolicus, A. mongolica*, and *P. mongolica*) and two narrow-range species (*T. mongolica* and *H. songaricum*) (Fig. 3). Stacking of five distribution models resulted in a map with centers of high suitability for the shrubs (Fig. 3a). Centers of high suitability were mainly located near Yellow River around Wuhai City. Additional hotspots were found in southwestern Zhongwei. A narrow area in northwestern Yellow River was identified as highly suitable for most taxa (Fig. 3a). In an attempt to visualize suitable climate space within human disturbance gradient, the model that identified a suitable space was mapped in dimensions of human footprint and population density (Supplementary Fig. 1). The suitable space for *A. mongolicus* and *A. mongolica* fell into areas of both high and low human disturbances, whereas *P. mongolica* tended to occupy areas of low human disturbance. For the two narrow-range species, *T. mongolica* can survive in areas with low to medium population density, whereas *H. songaricum* tended to occupy in sparsely populated region, both the two taxa tended to occupy areas of low human disturbance.

### Ecological niche properties

Analysis of ecological niche properties rendered a PCA with the first axis mainly loaded by GDD, AI, slope, and NDVI, which explain 30.6% of total variation (Fig. 4). The second axis explained about 14.6% of the variation and was loaded by SQ1, SQ3, and BIO6.

Results from niche breadth assessment showed a high variation in environmental suitability for relic shrubs (Supplementary Table 1). The highest niche breadth that we found was *A. mongolica* (0.1109), which presented the broadest distribution of suitable habitat (Fig. 3). Two other wide-range species (i.e. *A. mongolicus* and *P. mongolica*) also exhibited broad niche breadth. *T. mongolica* (0.0228) and *H. songaricum* (0.0075) exhibited narrow niche breadth, corresponding to their limited geographic distributions (Supplementary Table 1, Fig. 3). Niche overlap results suggest high variations in environmental space inhabited by different shrubs (Table 2, Fig. 4). Great overlaps were observed between *A. mongolicus* and *A. mongolica* (0.585) and between *H. songaricum* and *T. mongolica* (0.484). However, the niche overlaps among other pairs were extremely low, ranging from 0.078 to 0.266, suggesting they occupy considerably different environment niches. Even in comparisons between wide-range species, niche overlaps (e.g. between *A. mongolicus* and *P. mongolica*, and between *A. mongolica* and *P. mongolica*) were extremely low (Table 2), these species differed in their occupied niche spaces (Fig. 4). All niche overlap values are presented in Table 2.

Null hypothesis of niche equivalency test was rejected for all comparisons between the five shrubs, except between *A. mongolicus* and *A. mongolica* (Table 2). By contrast, in our analysis of niche similarity, the null hypothesis held for all pairs of shrubs (i.e. niche similarity in Table 2). For comparison between *A. mongolicus* and *A. mongolica*, niche equivalency was supported, whereas niche similarity was rejected. Niche overlap between *A. mongolicus* and *A. mongolica* was high (Supplementary Table 1), corresponding to niche equivalency test.

## Discussion

Identification of main environmental constraints on species distribution is important for conservation actions of investigate future climate change effects^1^. In this study, we identified biodiversity hot spots and key environmental constraints on distributions of five relic shrubs by mapping ecological dimensions and potential distributions using state-of-the-art ENM and ordination techniques. The wide distribution of relic shrubs in western Ordos plateau biodiversity center underlines the variety of environmental conditions in which they are adapted, these conditions reflected physiological differences inherent in these shrubs.

### Environment shaping distribution

Environmental factors that shape shrub distribution varied considerably. Arid index is found to be the most important factor for *A. mongolicus* (Fig. 2). Here aridity index was used to quantify precipitation availability over atmospheric water demand^20^. In western Ordos, climate is extremely dry, the annual precipitation is about 272.3 mm. Our results suggest that precipitation is an important limiting factor for *A. mongolicus, A. mongolica*, and *T. mongolica*. These plants have developed high capability in water absorption and decrease in transpiration to expand their geographical ranges (e.g. *A. mongolicus*)^21^. A linear response to AI was observed in *A. mongolicus*, whereas unimodal and semi-unimodal responses were observed in *T. mongolica* and *A. mongolica*, respectively (Fig. 2). Slope steepness affects plant growth through differential incidence of solar radiation, wind velocity, or soil type. Xerophytic plants are known to inhabit the south-facing slope^22^. Here, slope was found to be an important factor in three wide-range species. Less exposure to insulation and moisture abundance in soil probably be important to these species. For *H. songaricum*, soil quality (i.e. SQ1 and SQ3) was found to be the most important factors. *H. songaricum* is a deciduous shrub, this plant is found primarily on lithoid hillsides (about 1000 m to 1300 m) in western Ordos. Su *et al*. suggested that the species dispersed from Central Asia into western Ordos plateau through Hexi Corridor during Tertiary period^23^. Our results suggest that soil properties, including soil texture, bulk density, coarse fragments, and soil organic carbon (SQ1 and SQ3), mainly restricted present distribution of *H. songaricum* in western Ordos. As founding above, variables reflecting precipitation availability and soil quality characteristics usually play an important role in these relic shrubs. These findings will not only help us in understanding the present distribution, but also pave the way for investigations of species responses to climate change and future conservation actions^1^.

### Potential distribution

Given wide variation in environmental conditions in which shrubs grow, low niche overlap between these shrubs was expected. The low values of niche overlap were also reflected in their different environmental constraints. Geographical distributions of shrubs were generally consistent with their niche breadth because a broad niche allows species to persist in a wide range of habitat (Supplementary Table 1, Fig. 3). By contrast, a narrow niche restricts a species into the few places^24^,^25^. The limited distribution of *T. mongolica* and *H. songaricum* might be primarily due to their narrow niche breadth. Nonetheless, our results of ENM predictions showed that much suitable environmental space existed beyond known distributions of five shrubs, these areas might be useful in future investigation or transplantation actions. Biodiversity hotspots identified for five shrubs were mainly found around Yellow River in western Ordos, specifically in Wuhai area, southwestern Zhongwei, and a narrow area along northwestern Yellow River (Fig. 3). However, these areas fell into intensive human residence region bearing high human disturbance (Supplementary Fig. 1). In western Ordos, the human activities caused by urbanization or mining have seriously impacted relic shrubs^17^.

### Implications for conservation

Importance of relic shrubs in western Ordos were because of their floristics in plant evolution and biogeography, and their ecological function against desertification. Shrubs in Ordos plateau can resist wind, stabilize sand, preserve biodiversity, and protect habitats in certain degrees^17^. Notably, biodiversity conservation, ecological function and economic development are correlated and equally important in western Ordos, nonetheless this reality was not fully recognized by regional government, although a nature reserve has been set up for these species and other rare and endangered plants in western Ordos. Our results suggest that each relic shrub in western Ordos plateau is constrained by a unique set of environmental conditions, their non-equivalence of ecological niches implied that the distribution of one species cannot be implied by the distribution of another, highlighting the inadequacy of one-fits-all type of conservation measure. Conservation for each shrub should designed carefully to reflect its unique environmental requirements. Both *T. mongolica* and *H. songaricum* have declined drastically over the past decades and are classified as endangered species in China Species Red List^26^. Much suitable ecological space existed beyond known distributions of the five shrubs. However, these areas fell into areas of human activities and were severely affected by urbanization or mining. Thus, efforts to balance habitat protection and economic development should be prioritized in western Ordos.

## Methods

### Species data

**Figure 1:**
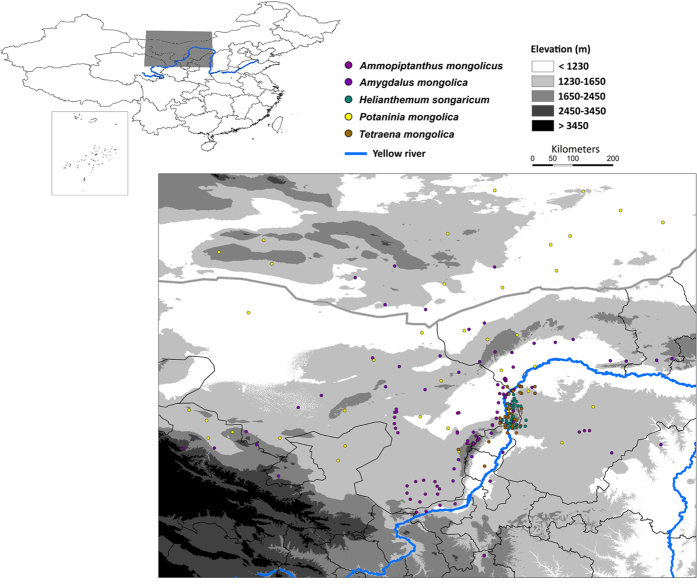
Study area. Figures show the location of the western Ordos plateau in China, together with occurrence points used in present study. Maps were generated in ArcGIS 10 (Environmental Systems Research Institute, www.esri.com).

**Figure 2:**
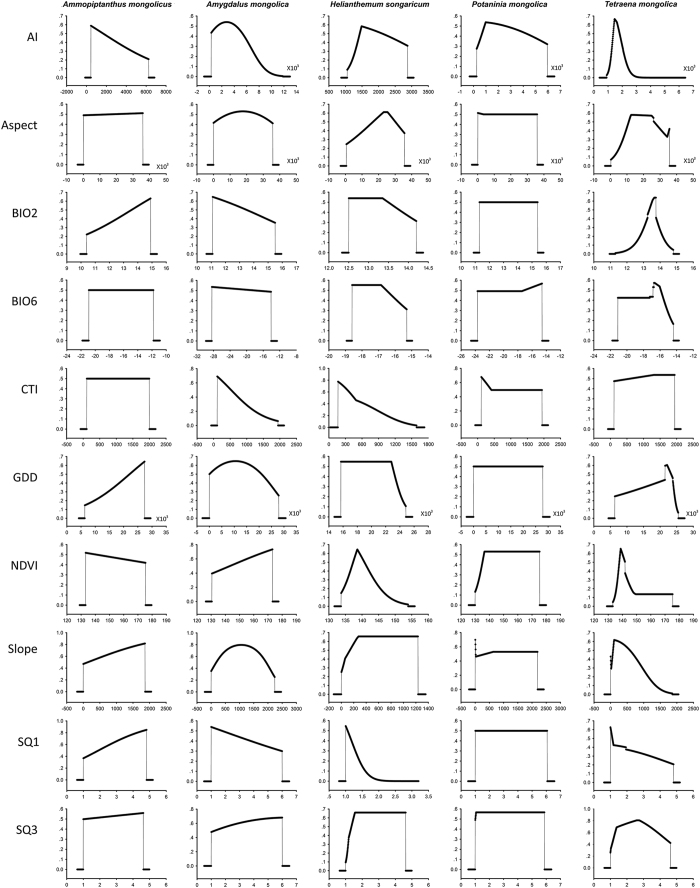
Environmental response curves for *Ammopiptanthus mongolicus, Amygdalus mongolica, Helianthemum songaricum*, Potaninia mongolica, and *Tetraena mongolica*. The x-axis of the variables represents their ranges for the complete study area, while the y-axis represents the predicted suitability of focus species. Variables abbreviations accord to descriptions in the text.

**Figure 3:**
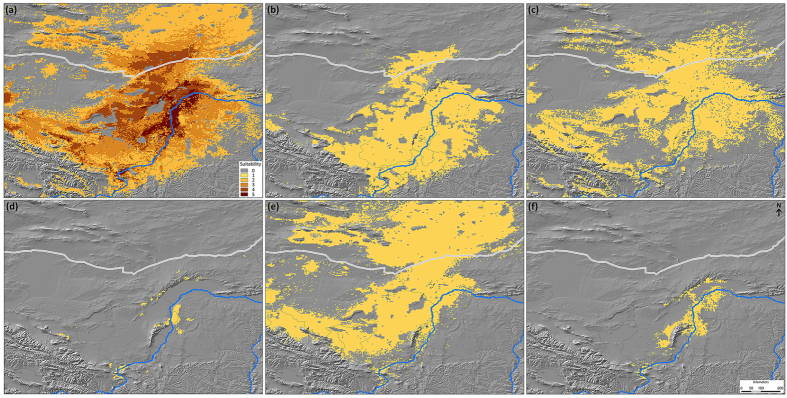
Potential distributions of relic shrubs in western Ordos. Panel (**a**) presents the results from an ensemble of five niche models, highlighting regions of shared habitat suitability. For panels (**b**–**f**), the yellowish brown represents the areas that have high habitat suitability after applying 10th training threshold ((**b**) *Ammopiptanthus mongolicus*, (**c**) *Amygdalus mongolica*, (**d**) *Helianthemum songaricum*, (**e**) *Potaninia mongolica*, (**f**) *Tetraena mongolica*). Niche model results were modified in ArcGIS 10 (Environmental Systems Research Institute, www.esri.com).

**Figure 4:**
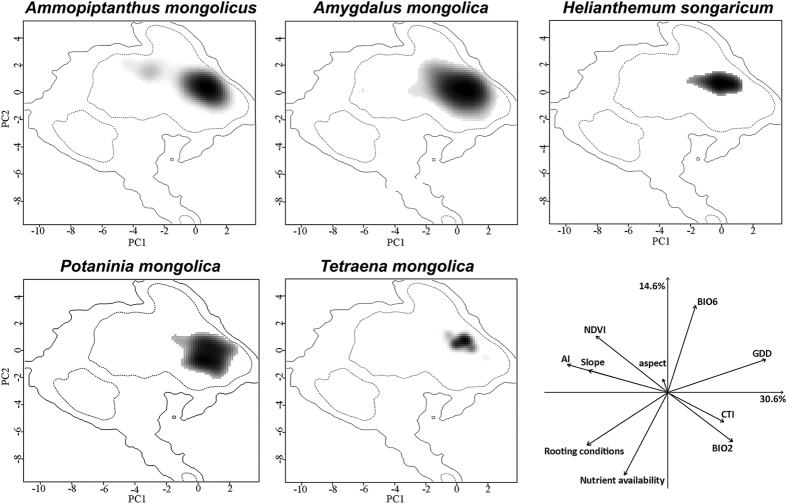
Ecological niche of five relic shrubs in environmental space. Niche were displayed in the two main axes of principal component analysis. Grey-to-black shading represents grid cell density of species’ occurrences (black being the highest density), the first dashed line represents the 50% of the available environment and the solid line represents the 100%. Last panel presents the contribution of variables for loading PCA_env axes and percentage of inertia explained by axes one and two.

Occurrence records were assembled from Global Biodiversity Information Facility Data Portal (www.data.gbif.org, accessed March 2014), Chinese Virtual Herbarium (www.cvh.org.cn, accessed March 2014), published studies^23^,^27^,^28^, and our field works in April 2013 and June 2014 (Fig. 1b, Supplementary Table 1). Geographic coordinates were denoted for each occurrence record. Spatial clusters of localities was eliminated^14^,^29^, we spatially rarefy our occurrence records by reducing occurrence localities to a single point within specified Euclidian distance in SDM toolbox^30^, which is particularly useful for studies with limited occurrence records. Study area was delimited to 99–11° E, 36–45° N based on geographical ranges of relic shrubs and location of Ordos biodiversity center^17^,^31^.

### Environmental data

Eighteen environmental variables [consisting of 11 climate variables, 4 topographic variables, 2 soil prosperity variables, and NDVI] were considered. Eight bioclimatic variables [annual mean temperature (BIO1), BIO2, maximum temperature of warmest month (BIO5), BIO6, annual precipitation (BIO12), precipitation of wettest month (BIO13), precipitation of driest month (BIO14), and annual mean radiation (BIO20)] represented annual trends and extreme conditions were considered, which were obtained from WorldClim^32^. Additionally, the GDDs above 5 °C were calculated following Synes and Osborne^33^; potential evapotranspiration (PET) and AI were derived from Consultative Group on International Agricultural Research (www.csi.cgiar.org, accessed March 2014). Topographic variables represented by elevation, slope, aspect, and CTI were derived from US Geological Survey^34^. Soil characteristics represented by SQ1, and SQ3 were obtained from Harmonized World Soil Database (www.iiasa.ac.at, accessed March 2014). NDVI was included because this parameter has been shown to increase accuracy of model prediction for vegetation mapping^35^,^36^. We finally selected 10 variables (AI, Aspect, BIO2, BIO6, CTI, GDD, NDVI, Slope, SQ1, and SQ3; Table 1) by presenting Pearson’s correlation ≤0.70^37^ at a 2.5 min resolution for analysis.

### Mapping distribution and ecological dimension

We used ENMs to analyze spatial distribution of relic shrubs and identify key environmental variables constraining their distributions. Maximum entropy modeling implemented in Maxent was adopted^38^, Maxent follows the principle of maximum entropy and distributes probability as uniformly as possible. Recent studies have shown great advancements on geographic background selection, and reduction of space correlation or model complexity in ENM^39^,^40^,^41^. We used SDM tool^30^ to prepare background bias file, as well as to fine tune model feature and regularization parameters for each species to best model their ecological niche and potential distribution. Model strength was estimated using area under the curve (AUC) of receiver operator characteristic (ROC) generated by Maxent. Null model approach^42^ was used to test significance of our model predictions. Added advantage of testing against a null model is that all collection localities can be used for model calibration^42^.

The relic shrub models were projected on study area to identify suitable habitats for their distribution and conservation. Model predictions were thresholded to produce binary maps using 10th training presence threshold, this approach is conservative in ecological niches estimation because this technique eliminates extreme values that may result from erroneous identification or georeference (i.e. partial ROC approach)^43^. Variables in models capable of predicting shrubs’ presence were identified through permutation importance test, a high percentage of permutation importance indicates a large relative decrease in AUC value after random permutation, thereby signifying high reliance on the variable^44^.

### Niche breadth, overlap, equivalency, and similarity

Niche breadth was estimated by applying inverse concentration measure of Levins as implemented in ENMTools^5^,^45^, we obtained niche breadth of each species to assess degree of shared niche space between shrubs. Niche breadth ranges from 0 (when all but one grid cell exhibits non-zero suitability) to 1 (when all the grid cells in the study area are equally suitable)^46^, species with a wider environmental distribution render higher niche breadth values. Assessment of niche overlap allows quantification of niche shared by the shrubs, niche overlap between pairs of shrubs was computed using Schoener’s *D* statistics^47^,^48^, ranging from 0 (when two species present no overlap in environmental space) to 1 (when two species share same environmental space). Niche equivalency and similarity were determined between pairs of shrubs using a kernel smoothing script^6^. Multivariate niche overlaps in gridded environmental space between pairs of taxa were compared using PCA_env function (i.e. principal component analysis on entire environmental space of the two ranges)^6^. Niche overlap was measured along the gradients of multivariate analysis; furthermore, statistical tests of niche equivalency and similarity^48^ were computed from the density in environmental space as described by Broennimann *et al*.^6^.

### Human disturbance

In an attempt to visualize human disturbances under which models identified suitability across western Ordos Plateau, human disturbance variables associated with model prediction were extracted and visually displayed in scatter plots. In this step, human disturbances were represented by human footprint and population density. The former is a composite summary of human influence on land surfaces^49^, whereas the latter is a gridded population distribution, which was obtained from Oak Ridge National Laboratory^49^.

## Additional Information

**How to cite this article**: Zhu, G.-P. *et al*. Mapping the ecological dimensions and potential distributions of endangered relic shrubs in western Ordos biodiversity center. *Sci. Rep.* **6**, 26268; doi: 10.1038/srep26268 (2016).

## Supplementary Material

Supplementary Information

## Figures and Tables

**Table 1:** Percentage of variable contribution to the model construction, derived from the permutation importance analysis.

**Variables**	***A. mongolicus***	***A. mongolica***	***H. songaricum***	***P. mongolica***	***T. mongolica***
Mean diurnal range (BIO2)	0	6.8	**6.5**	0	6.2
Maximum temperature of warmest month (BIO5)	6.3	3.1	0	2.9	9
Aridity index (AI)	**58.9**	**33.1**	1.8	9.7	**36.7**
Aspect	0	3.2	3.1	2.4	8.9
Compound topographic index (CTI)	0.2	0	0	4.8	0.1
Growing degree days (GDD)	0	**14.3**	0	0	6.2
Normalized Difference Vegetation Index (NDVI)	0	7.3	3.4	**45.7**	**10.3**
Slope	**20.3**	**27.5**	0	**22.2**	2.5
Nutrient availability (SQ1)	**14.2**	0	**67.5**	0	7.8
Rooting conditions (SQ3)	0	4.8	**17.6**	**12.3**	**12.2**

For each shrub, the three variables with the highest contributions are presented in bold.

**Table 2:** Niche comparisons between pairs of shrubs.

**Species** ***a***	**Species*****b***	**Niche overlap**	**Similarity**	**Equivalency**
*A. mongolicus*	*A. mongolica*	0.585	ns	Equivalency
	*H. songaricum*	0.078	Similar*	Different*
	*P. mongolica*	0.148	Similar*	Different*
	*T. mongolica*	0.086	Similar*	Different*
*A. mongolica*	*H. songaricum*	0.133	Similar*	Different*
	*P. mongolica*	0.231	Similar*	Different*
	*T. mongolica*	0.107	Similar*	Different*
*H. songaricum*	*P. mongolica*	0.266	Similar*	Different*
	*T. mongolica*	0.484	Similar*	Different*
*P. mongolica*	*T. mongolica*	0.253	Similar*	Different*

Niche overlap values are presented for the comparisons of niche similarity and equivalency of species *a* with species *b*.

^*^Ecological niches are significantly (*P* < 0.05) more similar or different than expected by random. ns: not significantly different.
