# Supplementary material for: Mapping the ecological dimensions and potential distributions of endangered relic shrubs in western Ordos biodiversity center
Source: Sci Rep. 2016 May 20;6:26268. doi: 10.1038/srep26268 (PMC4873805; doi:10.1038/srep26268)

**Supplementary information**

**Mapping the ecological dimensions and potential distributions of endangered relic shrubs in western Ordos biodiversity center**

Geng-Ping Zhu, Hui-Qi Li, Li Zhao, Liang Man, Qiang Liu

**Supplementary Table 1. Ecological niche models evaluation by their AUC and null model results**

| **Shrubs** | **n** | **AUC*** | **Null model AUC** | **Niche breadth** |
| --- | --- | --- | --- | --- |
| ***A. mongolicus*** | 56 | 0.90* | 0.70 | 0.1056 |
| ***A. mongolica*** | 39 | 0.79* | 0.72 | 0.1109 |
| ***H. songaricum*** | 20 | 0.99* | 0.79 | 0.0075 |
| ***P. mongolica*** | 35 | 0.72 | 0.74 | 0.1104 |
| ***T. mongolica*** | 34 | 0.98* | 0.74 | 0.0228 |

n, number of sample locations used for modelling the species distributions.

*Models have a significantly higher AUC value when compared to their null distribution (*P* < 0.01) based on 99 repetitions (mean of the null model score presented).

**Supplementary Figure 1.** Visualization of suitable and unsuitable areas of five relic shrubs in the dimensions of human footprint and population density across western Ordos Plateau. Areas of suitable and unsuitable for the shrubs were based on a 10% omission threshold.


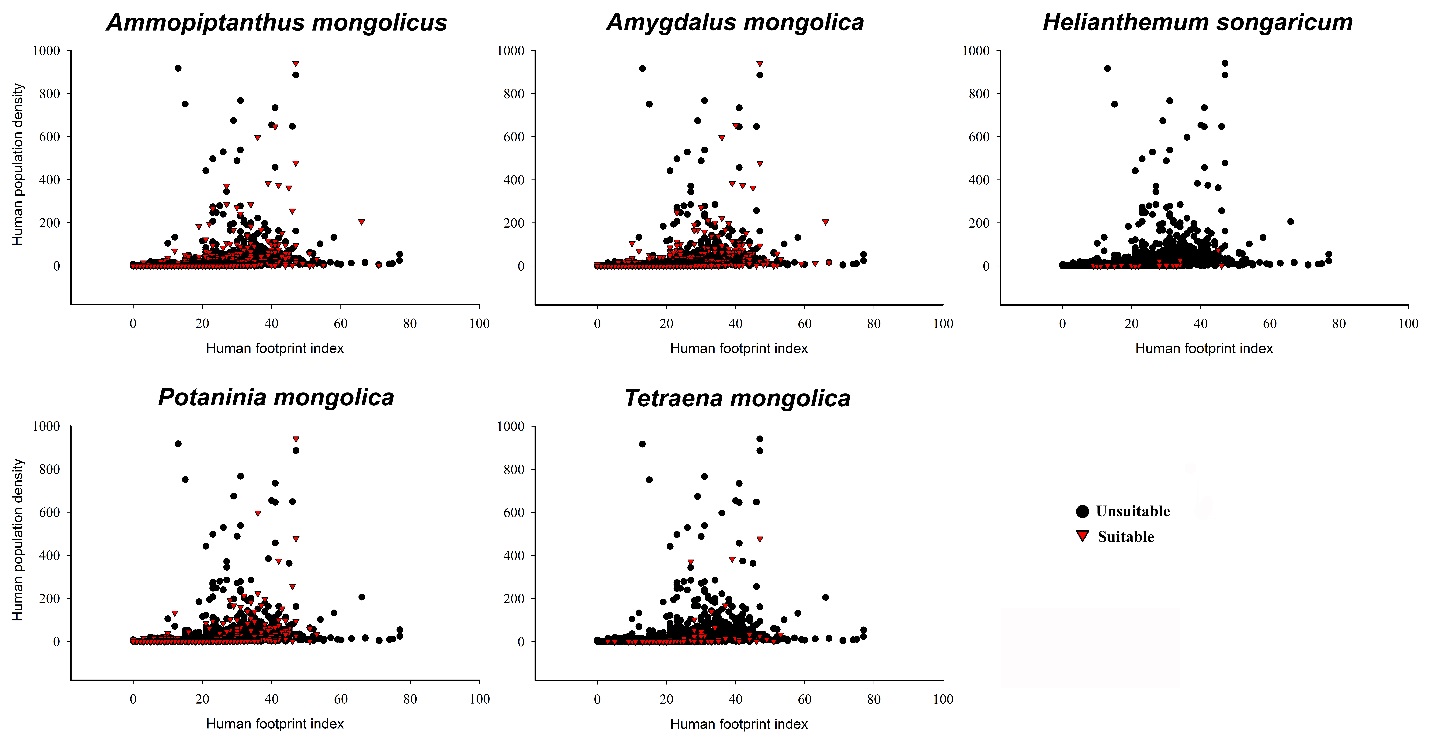

Supplement: Supplementary Information [file srep26268-s1.doc]
